# Highlighting the Undetectable — Fluorescence Molecular Imaging in Gastrointestinal Endoscopy

**DOI:** 10.1007/s11307-022-01741-1

**Published:** 2022-06-28

**Authors:** Judith A. Stibbe, Petra Hoogland, Friso B. Achterberg, Derek R. Holman, Raoul S. Sojwal, Jacobus Burggraaf, Alexander L. Vahrmeijer, Wouter B. Nagengast, Stephan Rogalla

**Affiliations:** 1 Department of Surgery, Leiden University Medical Center, Leiden University, Leiden, The Netherlands; 2 Department of Gastroenterology and Hepatology, University Medical Center Groningen, University of Groningen, Groningen, The Netherlands; 3 Department of Medicine, Division of Gastroenterology, Stanford University School of Medicine, Stanford, CA USA; 4 Centre for Human Drug Research, Leiden, The Netherlands

**Keywords:** Gastrointestinal endoscopy, Cancer, Inflammation, Early detection, Targeted biopsy, Fluorescence, Near-infrared fluorescence, Optical imaging, Molecular imaging, Fluorescence molecular endoscopy, Personalized medicine, Artificial intelligence

## Abstract

**Abstract:**

Flexible high-definition white-light endoscopy is the current gold standard in screening for cancer and its precursor lesions in the gastrointestinal tract. However, miss rates are high, especially in populations at high risk for developing gastrointestinal cancer (e.g., inflammatory bowel disease, Lynch syndrome, or Barrett’s esophagus) where lesions tend to be flat and subtle. Fluorescence molecular endoscopy (FME) enables intraluminal visualization of (pre)malignant lesions based on specific biomolecular features rather than morphology by using fluorescently labeled molecular probes that bind to specific molecular targets. This strategy has the potential to serve as a valuable tool for the clinician to improve endoscopic lesion detection and real-time clinical decision-making. This narrative review presents an overview of recent advances in FME, focusing on probe development, techniques, and clinical evidence. Future perspectives will also be addressed, such as the use of FME in patient stratification for targeted therapies and potential alliances with artificial intelligence.

**Key Messages:**

• Fluorescence molecular endoscopy is a relatively new technology that enables safe and real-time endoscopic lesion visualization based on specific molecular features rather than on morphology, thereby adding a layer of information to endoscopy, like in PET-CT imaging.

• Recently the transition from preclinical to clinical studies has been made, with promising results regarding enhancing detection of flat and subtle lesions in the colon and esophagus. However, clinical evidence needs to be strengthened by larger patient studies with stratified study designs.

• In the future fluorescence molecular endoscopy could serve as a valuable tool in clinical workflows to improve detection in high-risk populations like patients with Barrett’s esophagus, Lynch syndrome, and inflammatory bowel syndrome, where flat and subtle lesions tend to be malignant up to five times more often.

• Fluorescence molecular endoscopy has the potential to assess therapy responsiveness in vivo for targeted therapies, thereby playing a role in personalizing medicine.

• To further reduce high miss rates due to human and technical factors, joint application of artificial intelligence and fluorescence molecular endoscopy are likely to generate added value.

## Introduction

Every year around 3.6 million people worldwide are diagnosed with cancer of the upper or lower gastrointestinal (GI) tract, resulting in nearly 2.2 million deaths annually [1]. Early detection of (pre)malignant conditions is key to improving patient prognosis. Most GI cancers are preceded by slowly progressing precancerous dysplastic conditions, providing a window for effective intervention [2]. Intraluminal high-definition white-light endoscopy (WLE) with flexible endoscopes is the gold standard in the screening and surveillance of cancer in the GI tract. WLE focuses on detecting morphological features of (pre)malignant lesions; the diagnosis is subsequently confirmed by pathological analysis of obtained tissue biopsies. However, the miss rate of this approach remains high, especially with subtle premalignant lesions in high-risk patients. The miss rate of dysplastic epithelium in Barrett’s esophagus is reported to be 25% and miss rates as high as 28% are reported for (pre)malignant lesions in the lower GI tract in high-risk populations such as patients with inflammatory bowel disease (IBD) or Lynch syndrome [3–7]. In these patients, mucosal inflammation and metaplasia hamper the detection of small, flat, and subtle dysplastic lesions which tend to be malignant up to five times more often than the more common polypoid lesions [8, 9].

Considerable effort has been dedicated to the development of new imaging techniques to overcome this problem. Improving visualization of lesions based on their molecular features rather than morphology alone might aid in the early detection of lesions that are visually occult in white light. This technique is applied in fluorescence molecular imaging, which uses fluorescently labeled probes that bind to specific molecular structures or receptors expressed by (pre)malignant lesions and are made visible with dedicated light sources and camera systems. Incorporating this technique into flexible gastrointestinal endoscopy systems resulted in fluorescence molecular endoscopy (FME). In the last decade, research in the field has transitioned from preclinical to clinical studies, with promising results. Several early phase studies support FME as a successful way to detect (pre)malignant lesions, even before notable morphological changes appear [10–13]. Could this imaging strategy that highlights the undetectable be the solution to the current high miss rates?

In this narrative review, we will discuss the current status of FME in flexible gastrointestinal endoscopy (i.e., esophagogastroduodenoscopy and colonoscopy). We review current strategies including the selection of suitable molecular probes and available techniques and describe how they can be refined. We discuss the landmark clinical evidence, its gaps, and how these should be translated to clinical use. Finally, we address potential future applications of this promising diagnostic field, such as patient stratification for targeted therapies.

References for this review were identified by searching PubMed using the search terms “fluorescence,” “near-infrared fluorescence,” “optical imaging,” “molecular imaging,” “fluorescence molecular endoscopy,” “fluorescent tracer,” and “targeted fluorescent tracer.” Additionally, ClinicalTrials.gov and the Netherlands Trial Register were searched for ongoing clinical trials. References published on or before Sept 15, 2021 were considered. Articles were also identified through searches of the author’s files. Only papers published in the English language were reviewed. The final reference list was generated based on relevance to the broad scope of this Review.

## Molecular Probes Fit for Fluorescence Molecular Endoscopy

Before the development of targeted probes, fluorescence studies were predominantly performed with non-targeted tracers like the fluorescent probe Indocyanine Green (ICG). The mechanism of these tracers relies largely on the enhanced permeability and retention (EPR) effect, by which large-sized molecules or molecule-protein complexes accumulate in tumors due to their increased vascular permeability [14, 15]. Other probes like the heme precursor 5-aminolevulinic acid (5-ALA) rely on enhanced metabolism and accumulation of its fluorescent metabolite protoporphyrin IX in malignant tissue [16]. Selective uptake of 5-ALA by transporters also seems to play a role in the tumor environment; as a result this probe is already more target specific than tracers like ICG [17]. However, because inflammatory cells can manifest these same features as malignant cells, both 5-ALA and tracers relying on the EPR effect are not highly specific [18, 19]. Another strategy thoroughly studied in colonoscopy is autofluorescence imaging. It is based on the principle that endogenous tissue fluorophores such as collagen and hemoglobin emit fluorescent signals when subjected to light of a specific wavelength, and therefore are label free. Nevertheless, this method seems to have no major additional value for polyp detection and therefore has no place in current endoscopy guidelines [20–22]. Aiming to improve upon these preceding strategies, more recently fluorescent studies have used targeted probes that bind to specific molecular characteristics of (pre)cancerous lesions, the specific microenvironment or biological processes. Probe-to-target binding that is strong and highly specific increases target visualization by enhancing the contrast. However, implementing fluorescent molecular probes is challenging and requires multidisciplinary teams and standardized procedures for the integration of clinical workflows in GI endoscopy. We will review these topics in the following paragraphs.

### Target and Probe Selection

Strong fluorescence signal in the (pre)malignant target area compared to the surrounding healthy tissue increases the target-to-background or tumor-to-background ratio (TBR) and enhances visualization of the lesion. This enables taking image-guided biopsies, which will direct clinical decision-making in terms of whether resection of a lesion is required, or other therapies are needed if the agent binds to specific target tissue or lesions of interest. A target for molecular detection should therefore comply with one or more of the following relevant features: (1) it is overexpressed in dysplastic or malignant cells, (2) it is minimally expressed in benign or inflamed tissue surrounding the target area, (3) it is upregulated in tumor-associated cells or structures, or (4) it is activated by the microenvironment specifically belonging to the target area [23, 24]. When FME is used following tumor treatment, such as (neoadjuvant) chemoradiotherapy, it is important to be aware that these treatments might affect expression of the target or the surrounding tissue [25, 26]. Examples of targets used in FME are epidermal growth factor receptor (EGFR, overexpressed in colorectal cancer) and vascular endothelial growth factor A (VEGFA, present in early stages of colorectal neoplasms and Barrett’s dysplasia) [13, 23].

Selecting the appropriate molecular probe is of equal importance to target selection. Every probe has its own pharmacodynamic and pharmacokinetic profile, which affects biodistribution and tumor penetration. The half-life of probes generally correlates with their molecular size: the smaller the molecular size of the probe, the faster its distribution and accumulation in the targeted area and clearance from the body. In order of size, the most well-known available molecular probes investigated (pre)clinically are antibodies, antibody fragments, nanobodies, small molecules, and peptides. The dose-to-imaging interval needs to be well-balanced for each probe, because any circulating unbound probe may cause unwanted background fluorescence [27]. A probe with a longer dose-to-imaging interval, like antibodies, might be a disadvantage in the clinical workflow of endoscopic procedures. This is because an additional patient visit needs to be scheduled up to 3 days before the endoscopy for an intravenous administration of the imaging agent. Smaller probes like peptides have remarkably shorter dose-to-imaging intervals; however, developing such specific peptides is a complex process. It requires methods such as phage display, where the precise binding sites are often unknown [28]. General properties, advantages, and disadvantages of probe categories are summarized in Table 1. This table lists examples of probes and targets currently investigated in gastrointestinal FME, but also probes tested in abdominal fluorescence-guided surgery studies. FME has benefited from the pharmacological, safety, and imaging results obtained in these studies. For example, certain surgical studies discovered that using fragmented antibodies as a probe leads to faster distribution without losing specificity [36, 37]. These findings could be eligible for FME translation and should be studied more in-depth.

**Table 1:** Categories of molecular probes and their targets used in gastrointestinal imaging

Class	Examples of molecular probes	Target	Advantages	Disadvantages	Trial phase/field(s)	Applications
Antibodies	Bevacizumab-800C [12, 13, 23, 29, 30]	VEGFA	• Highly specific • High affinity to antigen • Long-lasting binding capacities •Drug visualization for evaluation of delivery and therapy response evaluation	• Potential immunogenicity (allergic reactions); low risk in non-therapeutic dose • Slow distribution in IV administration • Limited by receptor expression and heterogeneity of tumors	Clinical/endoscopy, surgery	• Detection of esophageal dysplasia (Barrett’s) • Detection of colorectal adenoma • Follow-up colorectal carcinoma • Therapy response evaluation/prediction
	Cetuximab-800CW [23, 31]	EGFR				
	SGM-101 [26, 32]	CEA				
	Adalimumab-FITC [33]	mTNFα				
	Vedolizumab-FITC [34]	α4β7 Integrin				
Fragmented antibodies	VB5-845D-800CW (anti-EpCam) [35, 36]	EpCAM	• Highly specific • High affinity to antigen • Faster distribution in IV administration due to smaller molecular size	• Still substantial interval between administration and imaging	Preclinical (clinical ongoing)/surgery	• Intraoperative detection of CRC
	8708 (ScFv)_2_-800CW 8709 ScFv-Fc-800CW [37]	EGFR				
Affinity peptides	RGD-ZW800-1 [38]	αvβ6 Integrin	•Fast distribution and elimination •Low immunogenicity	• Variable affinity • Difficult to develop	Clinical/endoscopy, surgery	• Intraoperative detection of CRC • Detection of esophageal dysplasia (Barrett’s) • Detection of colorectal adenomas
	EMI-137 [10, 39]	c-Met				
	KCCFPAQ [11]	V600E BRAF mutation				
	QRH*-Cy5 [40]	EGFR				
	KSP*-IRDye800 [40]	ErbB2				
Activatable probes	6QC-ICG [41]	Tumor microenvironment (activated by cysteine cathepsins)	• High target-to-background ratio due to specific activation	• Unclear toxicity profiles	Preclinical/surgery, endoscopy	• Detection of colorectal adenomas
Physiological substances	Folate-FITC [42]	Folate receptor-α	• Mostly safe in use due to physiological occurrence	• Limited use for molecular characterization • Phototoxicity of 5-ALA [44]	Clinical/surgery, endoscopy	• Intraoperative detection ovarian cancer • Detection of esophageal dysplasia (Barrett’s)
	5-Aminolevulinic acid (5-ALA) [43]	Intracellular porphyrin metabolism				
Nanoparticles	Surface-enhanced resonance Raman scattering nanoparticle (SERRS-NP) [45] Fluorescent silica nanoparticles (FSN) [46]	-	• Not relying on receptor expression, but to the enhanced permeability and retention (EPR) effect	• Potential (long-term) toxicity	Preclinical/endoscopy	• Detection of colorectal adenomas

*CRC* colorectal carcinoma, *IV* intravenous

### Route of Administration and Feasibility

It is relevant to consider the pharmacological and optical properties of individual targets and probes. For some probes, the previously mentioned disadvantages regarding distribution and clearance can potentially be overcome by direct topical application of the probe instead of intravenous administration [13, 39]. The probe is sprayed on the luminal surface of the entire colon or esophagus during the endoscopy and the unbound residue is rinsed off with water after a few minutes. This method no longer requires the additional patient visit and bypasses several other logistical challenges (e.g., clinical staff available for drug administration and room for the patient). Moreover, topical administration leads to lower systemic concentrations of the probe, reducing the risk of unwanted side effects and allergic reactions.

There are certain limitations to topical administration, as it requires spraying the entire surface to enable thorough examination. The limited size and relatively clean mucosal surface of the esophagus facilitate complete coverage; however, larger volumes of spray are needed for the larger colon. Prior to a colonoscopy, patients need to “clean” their colon thoroughly using laxatives, since fecal remnants and physiologically present mucus can impair mucosal coverage. Systemic administration, on the other hand, ensures a more even distribution of the probe throughout the tissue and allows the tracer to penetrate deeper, which may also display submucosal lesions. Furthermore, dosing is easier to standardize. Lastly, while the additional time required for topical probe administration may not be a burden to the patient, it could reduce the daily number of procedures. Thus, it has to be ensured that the benefits of topical application do not outweigh the potential advantages of systemic administration.

## Visualization of Molecular Probes and Targets

Besides selecting the most suitable molecular probe and the most viable way to administer it, other steps need to be taken to make the target visible. We will discuss how this is performed in current FME studies, as well as gaps in techniques and promising new strategies.

### Conjugated Fluorophores

**Fig. 1:**
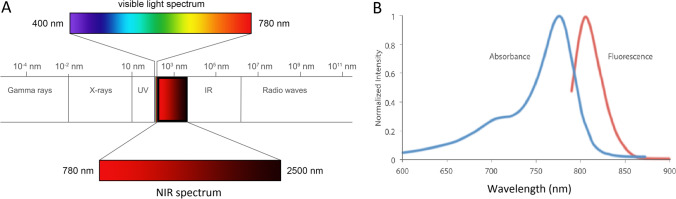
Light spectra and wavelengths. (**a**) The NIR spectrum lies between 780 and 2500 nm. Currently, almost all fluorescently labeled probes for FME are designed to emit in the NIR-I spectrum (780–900 nm). This design choice addresses three fundamental challenges: photon scattering by tissues, tissue autofluorescence, and tissue damage. First, the long wavelengths associated with both excitation and emission allow for deep-tissue imaging due to reduced scattering and increased penetration. Second: probes emitting in this spectral region benefit from high signal-to-background ratio, due to avoiding spectral regions associated with tissue autofluorescence. Third: the lower photon energies result in reduced tissue damage. (**b**) Example of excitation and emission spectra of the fluorescent dye IRDye 800CW. Due to vibrational relaxation in the excited or ground state orbitals, emitted photons must be equal to or lower in energy than the excitation photons. The emission spectrum is therefore red-shifted to longer wavelengths

In order to enable real-time and safe visualization, molecular probes are conjugated to a fluorescent dye — or fluorophore — which absorbs photons emitted by an external light source. Once a photon is absorbed, the fluorophore enters a state of excitation. Eventually, the fluorophore returns to its ground state, emitting the extra energy as light at a longer wavelength, creating a fluorescent signal [47, 48]. Currently, most fluorescent dyes used in FME studies emit in the near-infrared-I (NIR-I) spectrum, with a wavelength range from 780 to 900 nm (Fig. 1). This spectrum provides favorable properties for fluorescence imaging, as its longer wavelength allows for tissue penetration up to approximately 1 cm [49, 50]. Moreover, it reduces interference from autofluorescence whose excitation and emission wavelengths are mainly below 680 nm. Lastly, the fluorescence imaging at this wavelength does not interfere with the white light from the standard endoscope allowing the endoscopist to operate both white light and fluorescence simultaneously. More recently, fluorophores in the NIR-II spectrum (1000–1700 nm) have undergone preclinical testing. These fluorophores potentially improve image quality at deeper tissue levels due to increased penetration of the fluorescent signal [51]. Therefore, they could be of value in fluorescence-guided surgery, though there may be less benefit in flexible FME as most (pre)malignant lesions in the GI tract are located at the superficial mucosal layer. However, at present, it is not fully elucidated if wavelengths in the NIR-II spectrum are innocuous to tissues, and this should be studied further. We will focus on studies performed in the NIR-I spectrum further on in this review.

### NIR Endoscopy Systems

**Fig. 2:**
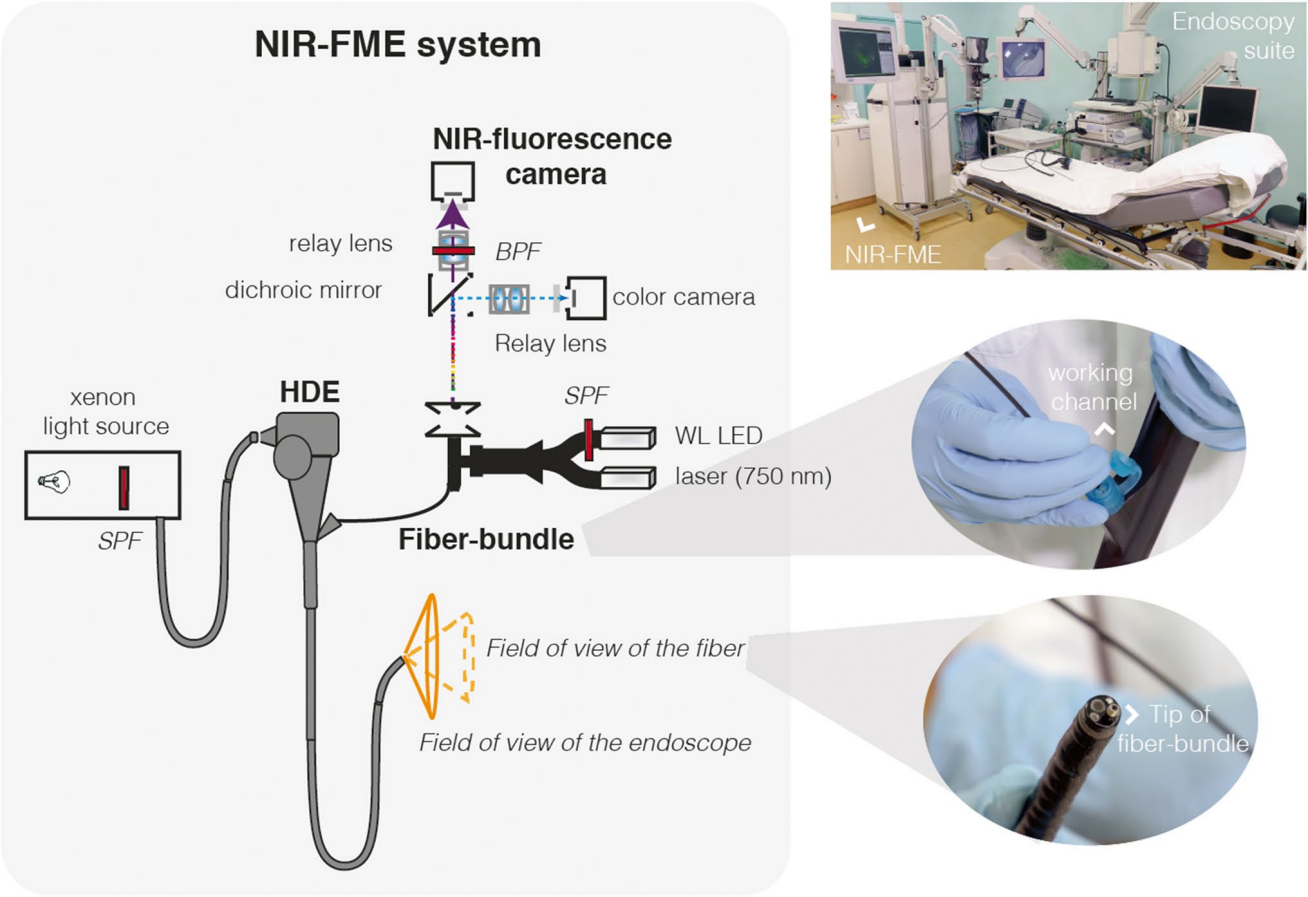
Schematic overview of a NIR-FME system. This figure illustrates the integration of a fiber bundle and an external NIR-fluorescence camera with a clinical endoscope. The NIR-system fiber bundle is inserted through the working channel of a standard clinical HD video endoscope (HDE). 750 nm laser light and short-pass filtered (SPF) white light from a LED are delivered through the illumination fibers of the fiber bundle to the distal end of the endoscope. Fluorophore-emitted and reflected white light return through the imaging fibers of the fiber bundle and are subsequently split by a dichroic mirror. Visible light is then detected by a color camera, and emitted fluorescent light is passed through a band-pass filter before being detected by an NIR-fluorescence camera. Previously published in Gut [13]

Visualizing the emitted fluorescent signal requires a dedicated NIR camera system to be incorporated in wide-field endoscopes. The standard charge-coupled device cameras are unable to translate the signal to the monitor due to their NIR filter systems. In contrast to surgical systems, the endoscopes used in GI endoscopy are flexible and long in order to be able to maneuver through the GI lumen (average length of 103–133 cm with an outer diameter of 8–12 mm). This long but narrow workspace complicates installation of the required optical hardware at the tip of the endoscope. Currently, there are no flexible NIR-imaging endoscopy systems on the market. Clinical studies are performed with modified fiber-based endoscopy systems, in which a fiber is inserted through the working channel of a conventional endoscope (mother-baby approach). This fiber conducts the excitation light to the endoluminal tissue of interest and leads the emitted signal back to a NIR camera system (Fig. 2). Although easy to apply and relatively cheap, a major disadvantage is the fact that the working channel is occupied by the fiber. Due to this, the working channel cannot be simultaneously used to guide the biopsy forceps to a lesion of interest after identification with FME. Switching gear through the working channel could lead to sampling error. This problem underlines the urgent need for the development of integrated wide-field endoscopy systems with detection and excitation filters for different wavelengths.

Unlike macroscopic wide-field endoscopy systems, confocal laser endomicroscopy (CLE) enables in vivo microscopic imaging of the intraluminal tissue with subcellular resolution. Tissue can be imaged with a thousand-fold magnification and tissue architecture can be evaluated during endoscopy [52]. Clinical decision-making can follow the physician’s histological assessment, on the spot, during endoscopy. By applying fluorescently labeled molecular probes and the required external light source, CLE can enable ad hoc assessment of lesions and cells based on their molecular signature, comparable to immunohistochemistry [28, 53]. This way, wide-field FME could serve as a macroscopic “red-flag” technique and consecutive CLE could provide microscopic information of the flagged lesion. CLE has shown promising results in clinical studies regarding dysplasia detection in Barrett’s esophagus, surveillance of colorectal polyps, and phenotyping of inflammation in IBD [52]. However, the microscopic images are generally only 300 × 300 µm, and peristaltic movements make it difficult to image and relocate the imaged area. Endoscopists also require additional training in interpretation of the microscopic images.

### Interpretation of Fluorescent Signals

“What you see is not always what you get”: as with many emerging imaging technologies, a combination of data preprocessing steps is required to correct for issues associated with data acquisition. Fluorescence intensity is influenced by multiple non-pathological variables, like absorption and scattering of light in tissue, or reflectance on the smooth surface of the mucus-covered mucosa. Altering the distance and angle of the endoscope to the tissue can significantly change the detected optical signal. The variable intensity might lead to incorrect interpretation, especially if the endoscopist is unaware of these confounding variables. Proper training in signal interpretation and imaging technique is critical, as well as standardized clinical workflows. Fluorescence quantification is a way to objectify the obtained signals. In most early FME studies, quantification was performed ex vivo with algorithms to account for differences in endoscope-tissue distance and geometry over the image field of view [10, 11, 13, 54, 55]. Although these methods could aid the endoscopist in correcting for some variables, a complete real-time correction is hard to achieve. Multi-diameter single-fiber reflectance (MDSFR) and single-fiber fluorescence (SFF) spectroscopy were developed to apply these corrections in vivo, in order to refine fluorescence quantification [29, 39, 56]. In these combinable techniques, the distal end of a fiber bundle is inserted through the working channel of the endoscope during endoscopy and placed onto the fluorescent lesion of interest. MDSFR spectroscopy measures signal absorption and scattering properties in tissue, while SFF spectroscopy measures tissue fluorescence. When combined, the fluorescent signal can be corrected by these optical properties, thereby allowing for quantification of fluorescence emitted by the fluorescent agent on the lesion of interest [56]. Although this is a promising technique which is successfully applied in multiple pilot studies, quantification is still based on post-procedural analysis and requires transitioning to real time to facilitate implementation in a clinical workflow [12, 29, 39, 57]. Additionally, because signal intensity can differ between different fluorescently labeled molecular probes, it would be helpful to determine signal thresholds for each fluorescent probe that can reliably predict whether a lesion is (pre)malignant [29].

## Clinical Evidence on Fluorescence Molecular Endoscopy in the Gastrointestinal Tract

Many probes in the NIR spectrum have been tested in preclinical settings for several purposes. In selected cases they made it through to patient studies, where they were found to be safe, feasible, and effective as well. We will illustrate the need for techniques to improve intraluminal lesion detection in the GI tract and discuss promising results of probes targeting these lesions.

### Fields of Interest for Fluorescence Molecular Endoscopy

Most studies on FME in the upper GI tract are performed in patients with Barrett’s esophagus. Barrett’s esophagus is a condition where the squamous epithelium of the esophagus is replaced with metaplastic columnar epithelium. Within this epithelium, precancerous dysplasia may arise. Because of this, Barrett’s esophagus is one of the most important risk factors for developing esophageal adenocarcinoma [58]. Although endoscopic surveillance programs have been set up for these patients, detection of dysplastic lesions with WLE remains challenging due to the often subtle morphological changes and patchy distribution. The current Seattle protocol recommends taking four-quadrant random biopsies every 2 cm, rather than only taking biopsies of visible lesions, to keep the miss rate as low as possible [59]. However, this method is prone to sampling error due to the fact that it is based on random biopsies, and because it is time-consuming resulting in low adherence to the protocol [60, 61]. Recent data shows that nearly 20% of endoscopists do not follow these guidelines for longer segments of affected Barrett’s esophagus [62]. This illustrates the urgent need for a more targeted approach, such as FME, in the surveillance of Barrett’s patients.

In the lower GI tract, the majority of FME studies are performed in the screening of colorectal cancer. This is one of the most common and lethal cancers worldwide, representing more than 9% of cancer-related deaths yearly [1]. Patients at a high risk for developing lower GI cancer, as in IBD, regularly undergo screening colonoscopies with the aim of early detection and timely intervention [63, 64]. However, the miss rate of dysplastic lesions is about three to five times higher in these patients compared with healthy individuals, as lesions are often non-polypoid (flat or non-pedunculated) [5, 6, 8, 9]. Moreover, lesions in IBD patients are often camouflaged against the background of inflamed or otherwise impaired mucosa. Therefore, an endoscopic surveillance modality such as FME that focuses on molecular features rather than on morphology alone could be of additional value for high-risk patients.

### Current Available Clinical Evidence on Lesion Detection

Several clinical trials have been conducted on FME with probes targeting (pre)malignant lesions of the GI tract. The current landmark studies are summarized in Table 2. As shown in this table, both affinity peptides and antibodies have been studied for enhancement of lesion detection in both patients with Barrett’s esophagus and patients at high risk for colorectal carcinoma. Burggraaf and colleagues performed one of the first patient studies, in which they paved the way for future research on this topic [10]. In this pilot study, the c-Met targeting peptide EMI-137 was administered intravenously 3 h prior to colonoscopy with NIR imaging and detected colorectal neoplasms that would otherwise remain unnoticed [10]. High TBRs were found, which were determined ex vivo with algorithms to correct for distance and geometry over the image field of view. In a related study that uses the same peptide, the initial findings regarding improved detection of colorectal neoplasms were confirmed [57]. Lower TBRs were found; however, these ratios were assessed in vivo by use of MDSFR/SFF spectroscopy. This underlines the importance of methods to correct for tissue absorbance and scattering properties for a more reliable interpretation of in vivo results. In addition, they performed subgroup analysis on different dose-to-imaging intervals from 3 h prior to endoscopy to 1 h, which showed no significant differences. This implies that a clinically favorable interval of 1 h preceding endoscopy could be applied in further studies.

**Table 2:** Landmark clinical evidence on fluorescence molecular endoscopy in the gastrointestinal tract

	Probe	Target	*N*	Aim of study				Route of admission		NIR system			In vivo fluorescence visualization		Quantification of intrinsic fluorescence		Subgroup analysis			Main outcomes
				Detection of dysplasia in BE	Detection of colorectal adenomas, high risk patients	Restaging LARC after nCRT	Assessing likeliness of therapy response in IBD	Topical	Systemic (IV)	Fiber-based FME	Multiplexed	CLE	After detection with WLE	Simultaneously with WLE	Ex vivo algorithms to correct for tissue variables	In vivo quantification with MDSFR/SFF	Topical vs. IV administration	Several administered doses	Several dose-to-imaging intervals	
Burggraaf et al. (2015) [10]	EMI-137	c-Met	15		X				X	X			X	X	X					-FME detected all 38 adenomas visible with WLE, and an additional 9 lesions that were not visible in WLE. Mean TBR 2.3 ± 1.1 -Also hyperplastic lesions and serrated polyps were visualized with FME -High fluorescence associated with c-Met expression
Joshi et al. (2017) [11]	KCC*-FITC	V600E BRAF mutation	38		X			X		X			X		X					-2.43-fold higher mean fluorescence intensity in SSAs compared to normal colonic mucosa -Differentiation of SSAs from normal mucosa with sensitivity of 89% and specificity of 92% at TBR of 1.16 -Higher mean fluorescence intensity of peptide bound to SSAs than bound to hyperplastic polyps in analysis of ex vivo specimens
Hartmans et al. (2018) [12]	Bevacizumab-800CW	VEGFA	17		X				X	X				X		X		X		-Colorectal adenomas showed high fluorescence -High concentrations of target in dysplastic areas (4.81–6.86 nmol/ml) compared to normal mucosa (3.73–3.82 nmol/ml) -Best results in 25-mg dose cohort compared to 4.5 mg and 10 mg, with 40% increase of intrinsic fluorescence (vs. 10 mg), mean TBR of 1.84 and detection of even small adenomas < 3 mm
Nagengast et al. (2019) [13]	Bevacizumab-800CW	VEGFA	14	X				X	X	X				X	X		X			-Overall detection enhancement of 25% compared with WLE and NBI -Topical application favorable over IV with higher TBRs (mean 4.30 vs. 2.75) and even 33% detection enhancement -High intracellular target staining congruent with fluorescence signals recorded ex vivo
De Jongh et al. (2020) [57]	EMI-137	c-Met	15		X				X	X				X		X			X	-Higher fluorescence in colorectal lesions than in surrounding tissue; TBR 1.53–1.74 -No clinically significant differences among various dose-to-imaging intervals (1–3 h), although 1-h interval preferred from a clinical perspective
De Jongh et al. (2020) [39]	EMI-137	c-Met	15	X				X	X	X				X		X	X	X		-Identification of 16/18 dysplastic lesions (89%); modest TBRs in Barrett’s segments (1.12–1.50) -c-MET membrane overexpression in 14/17 dysplastic lesions (82%) -Stomach-type epithelium also showed increased levels of c-Met membrane expression, complicating lesion detection in the distal esophagus
Tjalma et al. (2020) [29]	Bevacizumab-800CW	VEGFA	25			X			X	X				X		X				-Significant higher intrinsic fluorescence of tumor tissue compared to normal rectal tissue or fibrosis -Restaging with quantification of FME resulted in a positive predictive value and accuracy of 95% and 92% (87.5% and 84% for MRI, 90% and 80% for WLE) -FME with quantification would have changed diagnosis in 4 of 25 patients (16%)
Chen et al. (2021) [40]	QRH*-Cy5 KSP*-IRDye800	EGFR ErbB2	22	X				X		X	X		X		X					-92% of neoplastic lesions could be visualized, 11% false positives -Mean TBR’s for dysplasia/EAC 1.61 ± 0.21 and 1.68 ± 0.24 for QRH*-Cy5 and KSP*-IRDye800 resp. -High expression of EGFR and ErbB2 in high-grade dysplasia and EAC
Atreya et al. (2014) [33]	Adalimumab-FITC	mTNFα	25				X	X				X	X		X					-Intestinal mTNF + immune cells could be detected in vivo -Patients with high numbers of mTNF + cells showed significantly higher short-term response rates (92%) at week 12 upon subsequent anti-TNF therapy -Clinical response was sustained over a follow-up period of 1 year
Rath et al. (2017) [34]	Vedolizumab-FITC	α4β7	5				X		X			X	X							-Molecular imaging before therapy revealed pericryptal α4β7 + cells in the mucosa of patients who had sustained clinical and endoscopic response to subsequent therapy (*n* = 2)

*BE* Barrett’s esophagus, *EAC* esophageal adenocarcinoma, *EGFR* epithelial growth factor receptor, *ErbB2* epithelial growth factor receptor 2, *FAP* familiar adenomatous polyposis, *IV* intravenous, *LARC* locally advanced rectal carcinoma, *mTNFα* membrane-bound tumor necrosis factor alpha, *NBI* narrow-band imaging, *nCRT* neoadjuvant chemoradiotherapy, *SSA* sessile serrated adenomas, *TBR* target-to-background ratio, *VEGFA* vascular endothelial growth factor A, *WLE* white-light endoscopy

**Fig. 3:**
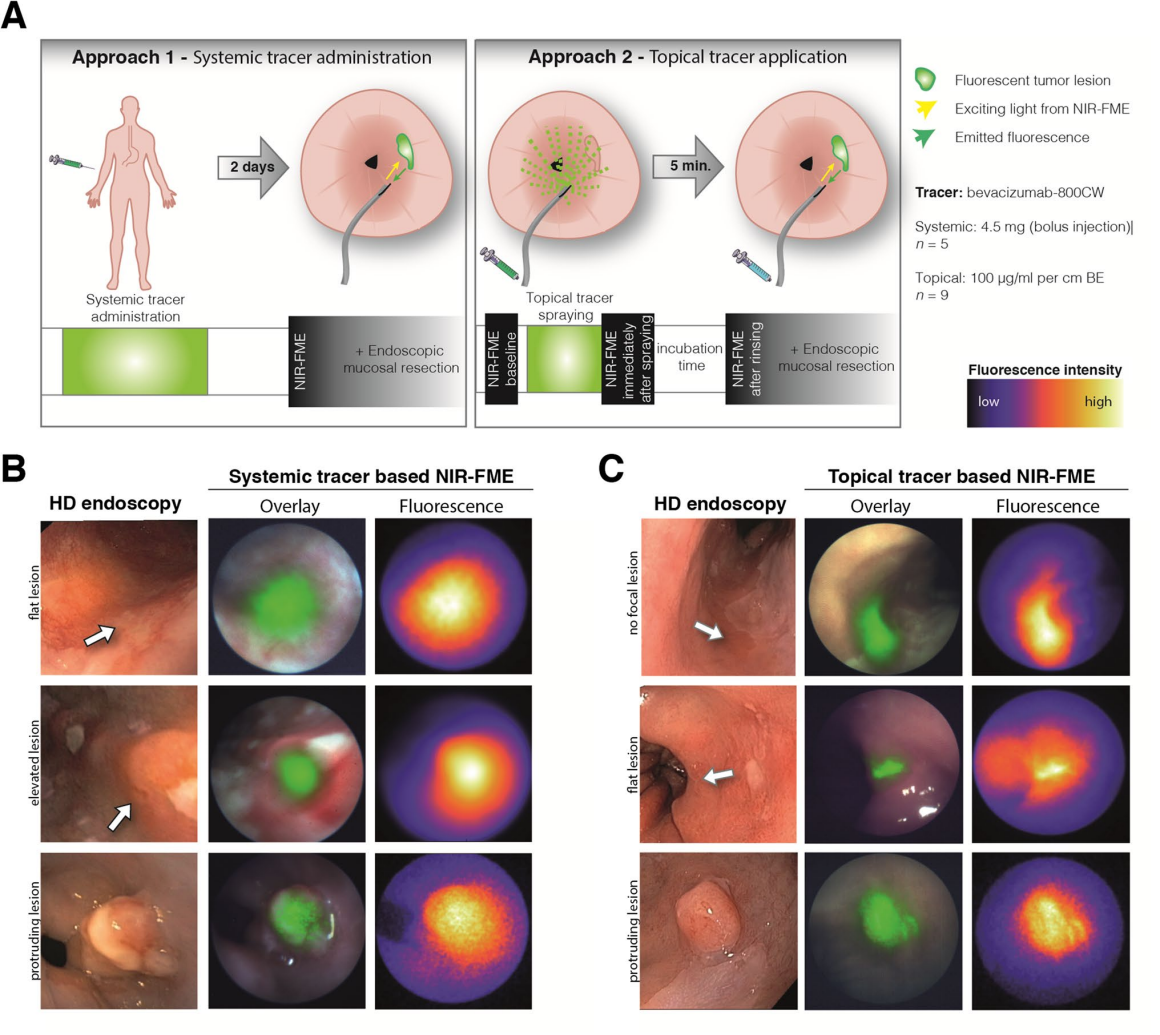
Overview of real-time VEGFA-targeted FME in Barrett’s esophagus. (**a**) Schematic overview and timeline of two NIR-FME approaches, i.e., intravenous administration and topical application. (**b**) Examples of results after intravenous administration of bevacizumab-800CW, and (**c**) results after topical application. The first row in Fig. 3c displays a lesion that was not visible during white light endoscopy but turned out to be adenocarcinoma. Previously published in Gut [13]

Nagengast and colleagues were one of the first to use FME to improve dysplasia detection over standard WLE in patients with Barrett’s esophagus. They administered the fluorescently labeled monoclonal antibody bevacizumab-800CW both topically and intravenously (2 days prior to endoscopy), which led to successful real-time visualization of dysplasia and adenocarcinoma (Fig. 3) [13]. The overall detection was improved by 25% over WLE and narrow-band imaging. Compared to intravenous administration, topical application resulted in favorable TBRs and enhanced detection by 33%. However, the sample size was small, with 14 patients, and TBRs were calculated ex vivo with algorithms. A larger phase II study in 60 patients is ongoing [30]. A similar study was performed with EMI-137. Administration of the tracer was switched from systemic to topical after an interim analysis of five patients where there were relatively low tracer concentrations in the lesions, leading to poor detection [39]. The quantified TBRs were modest; nevertheless, 89% of dysplastic lesions were identified correctly after topical application of the probe. However, stomach-type epithelium also showed increased levels of c-Met membrane expression, which complicates lesion detection in the distal esophagus where most neoplastic Barrett’s lesions are found [39]. Although this study shows that c-MET may not be the most ideal probe for lesion detection in Barrett’s esophagus, it is an excellent example of an iterative translational process where interim analysis affects study design.

A last noteworthy clinical trial on lesion detection in Barrett’s esophagus was recently published by Chen and colleagues. In this first-in-human study, a new technique of multimodal FME was performed, using two excitation lasers of different wavelengths (638 and 785 nm) guided through a single flexible fiber. With this multiplexed imaging tool and the topical application of two different fluorescently labeled peptides (QRH*-Cy5 specific for EGFR and KSP*-IRDye800 specific for ErbB2), 92% of the present neoplastic lesions were successfully visualized [40]. This study demonstrates the ability to simultaneously detect multiple targets in vivo, as well as detection of neoplasms that are molecularly heterogeneous.

### Towards Personalized Medicine

Besides enhancement of lesion detection, FME could also play a role in personalizing treatment strategies. This is illustrated in a clinical study by Tjalma and colleagues, using FME and spectroscopy with bevacizumab-800CW on restaging locally advanced rectal cancer after neoadjuvant chemoradiotherapy (nCRT) [29]. At present, nCRT is followed by surgical resection. However, in up to 27% of patients no residual cancer cells are found in the surgical specimen after nCRT; for example, they have a pathological complete response and surgery could potentially have been avoided to reduce morbidity and increase survival rates [67–70]. However, distinguishing residual tumor from fibrosis is challenging in WLE and MR imaging, which are the current standard restaging methods. Results of restaging with FME were compared with results of standard clinical restaging (MRI and WLE), and were correlated with the histopathology of the surgical specimen. FME with spectroscopy resulted in a higher positive predictive value and accuracy compared to MRI and standard endoscopy [29]. This suggests that implementing FME in restaging could lead to better stratification and potentially less undertreatment and overtreatment.

In vivo molecular characterization can also be used to evaluate drug delivery to targeted tissue and potentially predict therapy response. Goetz and colleagues conducted a preclinical study performing CLE with fluorescently labeled cetuximab: an antibody targeting EGFR, which is a component of the multimodal chemotherapy regimen in metastatic colorectal carcinoma. Human colorectal carcinoma cell lines were induced in mice and CLE was implemented before and after treatment with cetuximab. High fluorescence signal before treatment was related to significantly slower tumor progression, better overall survival, and better physical condition compared to low fluorescence signal [31]. This suggests that stronger fluorescence signal is related to increased presence of molecular targets for chemotherapy. The technique was translated to a clinical study where CLE was performed with fluorescently labeled adalimumab in patients with active Crohn’s disease, targeting mucosal TNFα (mTNFα). Patients with high counts of mTNFα-expressing immune cells prior to subsequent treatment showed a better clinical response to adalimumab compared to patients with low cell counts. This effect was sustained over a 1-year follow-up period [33]. A similar pilot study was performed in Crohn’s patients unresponsive to anti-TNF treatment using fluorescently labeled vedolizumab, a gut-selective monoclonal antibody directed towards the integrin α4β7 [71]. The mucosal cells of patients who responded well to vedolizumab showed significantly more fluorescence prior to therapy, compared to the non-responders that did not express any α4β7-positive fluorescence [34]. These results warranted the ongoing larger-sized clinical trial [72].

### Current Gaps in Clinical Evidence

The clinical findings mentioned above include current landmark studies performed with FME. Although promising and high in quality, these are proof-of-concept studies with small sample sizes. Moreover, study designs and outcome measures differ strongly. This makes interpreting and comparing studies of a certain probe hardly possible, let alone comparing different probes for a certain indication. For this reason, currently available clinical evidence need further validation with larger study populations and stratified study designs.

Another research gap is that no FME studies have been carried out in patients with active IBD. These patients have a high risk of developing colorectal carcinoma and encounter high miss rates due to the camouflaging effect of the inflamed background [8, 9]. Selecting a suitable FME probe for this population may be challenging, as it must distinguish (pre)malignant lesions from inflamed or scarred tissue which might have similar molecular features. Since potential targets could differ greatly from non-IBD patients in terms of receptors and heterogeneity, ex vivo studies on the molecular signatures of IBD are essential for enhancing accuracy in the predictive capabilities of a molecular target [73, 74]. Promising preclinical results on colorectal neoplasm detection in active IBD are derived from the fluorescently labeled cathepsin-activated probe 6QC-ICG, which enabled demarcation of premalignant GI lesions in a large animal model [41]. Being a “smart probe,” 6QC-ICG targets the tumor microenvironment as it is binding to system cathepsins which are highly abundant in tumor-associated macrophages and less in immune cells of benign or even inflamed mucosa [75]. Areas of dysplasia as small as 400 μm were successfully detected 12 to 18 h after an intravenous bolus dose in murine and human-scaled porcine models, and were clearly demarcated within inflamed and ulcerated mucosa. These preclinical results are promising for future clinical FME studies in patients with IBD who suffer from mucosal inflammation and are at high risk of progression to malignant lesions.

## Translation From Clinical Evidence to Clinical Use

The recent transition from preclinical to clinical studies has shown that FME is able to visualize subtle, macroscopically invisible, or uncertain lesions in the upper and lower GI tract that are regularly missed during conventional flexible white-light endoscopy. FME might therefore be a very promising tool in GI endoscopy, addressing the high miss rates of (pre)malignant lesions in both upper and lower GI tract, and improving early detection. Moreover, endoscopic interventional options are rapidly increasing. Currently, premalignant or early-stage GI cancer can often be removed endoscopically. The combination of reliable early detection of (pre)malignant lesions and minimally invasive removal yields an interesting field of action for oncological care.

Moreover, the increasing number of unique probes or drugs for different molecular targets offers a wide range of potential future applications. FME could help determine the molecular characteristics of malignant lesions or inflammation, thereby paving the way for personalized targeted therapy in gastroenterology. By using fluorescently labeled drugs as a molecular probe, drug distribution and pharmacodynamics can be visualized in vivo, allowing for the possibility of predicting drug responsiveness. As discussed, this would apply for patient stratification in IBD and oncological treatment, i.e., neoadjuvant therapy in several malignancies. It might help determine whether a patient is prone to respond to therapy or not. The ultimate goal would be to label different types of drugs with different fluorescent dyes, and visualize them in vivo with multispectral camera systems. This could help to identify the optimal treatment before administering it in a therapeutic dose, which improves patient stratification, safety, and (cost) efficiency. MDSFR/SFF spectroscopy could measure mucosal concentrations, for optimizing the dose of the selected treatment.

Some obstacles need to be addressed before FME can be implemented in clinical practice. The most important one is the potentially confounding effect of the human factor: all the additional information that FME and accompanying modalities offer makes interpretation more complex and leads to inter-observer variabilities. Adequate training of endoscopists is needed to benefit from the complementary input offered by FME. However, gaining experience takes time and may be costly. Furthermore, the attention span of the endoscopist — which can be lowered by distraction or tiredness — will still be of substantial influence on detection rates. Artificial intelligence (AI), and particularly deep learning, is increasingly used for computer-aided detection (CAD) of (pre)malignancies in endoscopic images. Multiple studies have shown that AI algorithms developed for image analysis in colonoscopy can successfully recognize (pre)malignant colonic lesions, as well as grade the inflammation status in IBD patients [76–81]. These results have already been translated to the clinic with the launch of the first commercially available AI system for colonoscopy in 2019 (GI Genius, Medtronic). Recently the first randomized controlled trial on the use of CAD in upper GI endoscopy was published, showing that miss rates of gastric neoplasms were significantly lower in patients where CAD was applied compared to standard care [82]. These promising achievements substantiate that AI will play a substantial role in future endoscopy.

A recent meta-analysis by Spadaccini and colleagues showed that CAD-assisted colonoscopy significantly improves adenoma detection rates compared to high-definition WLE and available strategies that increase mucosal visualization, such as chromoendoscopy [76]. However, CAD mainly depends on morphological features of lesions and requires excellent images. Therefore, it still depends on the endoscopic capabilities of the operator. Unlike colonic polyps, lesions that resemble the surrounding mucosa, as in Barrett’s dysplasia, are more difficult to detect using CAD and require even higher quality images [78]. At present, no data is available to assess the value of CAD for FME images; however, this should be explored. The combination could reduce human error and technical factors by standardizing recognition and interpretation of fluorescence images based on molecular structures, while deep learning networks continuously refine their output. These two forces combined could be of substantial benefit in the battle against high miss rates.

Another obstacle that needs to be addressed is the extra procedure time that FME requires due to administering of the probes and switching fibers and camera systems. If FME were used in all procedures, it could reduce the total number of operations by 1/5th (assuming that 5 min is added to every 20-min procedure). Therefore, technological refinement is required to streamline procedures. With integrated NIR systems — eventually with multiple spectra for simultaneous use of multiple tracers — FME could be efficiently applied in wide-field endoscopy without the need for switching fibers through the working channel. Moreover, in certain patient populations FME might reduce procedure time because fewer biopsies have to be taken. All in all, in every particular procedure the extra time that FME requires has to be balanced against the possible (time) gain it could give. Patients who are at high risk for (pre)cancerous lesions like patients with Lynch syndrome, IBD, and Barrett’s esophagus will benefit most — healthwise, costwise, and timewise.

## Conclusion

Fluorescence molecular endoscopy is a rapidly emerging field in flexible GI endoscopy that enables the visualization of lesions by detecting molecular changes rather than morphological changes. As molecular alterations in oncogenesis can appear before lesions become visible to “the naked eye,” FME can serve as a modality for early intraluminal detection of dysplastic lesions or GI cancer. It has the potential of improving screening programs for at-risk populations, as well as playing a part in personalizing medicine. Although work must be done to refine strategies and strengthen clinical evidence, we believe that FME might have an important role in GI endoscopy in the near future. Cooperation between clinicians, pharmacists, biologists, chemists, and engineers will give rise to this promising new imaging strategy in gastrointestinal endoscopy with great impact on both diagnostics and personalized medicine.

## Abbreviations

AI: Artificial intelligence
CAD: Computer-aided detection
CLE: Confocal laser endomicroscopy
EGFR: Epidermal growth factor receptor
EPR: Enhanced permeability and retention
FME: Fluorescence molecular endoscopy
GI: Gastrointestinal
IBD: Inflammatory bowel disease
ICG: Indocyanine Green
LED: Light-emitting diode
MDSFR: Multi-diameter single-fiber reflectance
MRI: Magnetic resonance imaging
mTNFα: Mucosal tumor necrosis factor alpha
nCRT: Neoadjuvant chemoradiotherapy
NIR: Near-infrared
SFF: Single-fiber fluorescence
TBR: Target-to-background ratio/tumor-to-background ratio
VEGFA: Vascular endothelial growth factor A
WLE: White-light endoscopy
5-ALA: 5-Aminolevulinic acid

## References

[1] Ferlay J, Colombet M, Soerjomataram I, Parkin DM, Piñeros M, Znaor A et al (2021) Cancer statistics for the year 2020: an overview. Int J Cancer. 10.1002/ijc.33588 10.1002/ijc.33588 33818764

[2] Săftoiu A, Hassan C, Areia M, Bhutani MS, Bisschops R, Bories E (2020). Role of gastrointestinal endoscopy in the screening of digestive tract cancers in Europe: European Society of Gastrointestinal Endoscopy (ESGE) Position Statement. Endoscopy.

[3] Braden B, Jones-Morris E (2018). How to get the most out of costly Barrett’s oesophagus surveillance. Dig Liver Dis.

[4] Visrodia K, Singh S, Krishnamoorthi R, Ahlquist DA, Wang KK, Iyer PG et al (2016) Magnitude of missed esophageal adenocarcinoma after Barrett’s esophagus diagnosis: a systematic review and meta-analysis. Gastroenterology 150(3):599–607.e7; quiz e14–5 10.1053/j.gastro.2015.11.040 PMC4919075 26619962

[5] van Rijn JC, Reitsma JB, Stoker J, Bossuyt PM, van Deventer SJ, Dekker E (2006). Polyp miss rate determined by tandem colonoscopy: a systematic review. Am J Gastroenterol.

[6] Leufkens AM, van Oijen MG, Vleggaar FP, Siersema PD (2012). Factors influencing the miss rate of polyps in a back-to-back colonoscopy study. Endoscopy.

[7] Stoffel EM, Turgeon DK, Stockwell DH, Zhao L, Normolle DP, Tuck MK (2008). Missed adenomas during colonoscopic surveillance in individuals with Lynch syndrome (hereditary nonpolyposis colorectal cancer). Cancer Prev Res (Phila).

[8] Soetikno R, Subramanian V, Kaltenbach T, Rouse RV, Sanduleanu S, Suzuki N et al (2013) The detection of nonpolypoid (flat and depressed) colorectal neoplasms in patients with inflammatory bowel disease. Gastroenterology 144(7):1349–52, 52.e1–6 10.1053/j.gastro.2013.04.008 23583483

[9] Soetikno RM, Kaltenbach T, Rouse RV, Park W, Maheshwari A, Sato T (2008). Prevalence of nonpolypoid (flat and depressed) colorectal neoplasms in asymptomatic and symptomatic adults. JAMA.

[10] Burggraaf J, Kamerling IM, Gordon PB, Schrier L, de Kam ML, Kales AJ (2015). Detection of colorectal polyps in humans using an intravenously administered fluorescent peptide targeted against c-Met. Nat Med.

[11] Joshi BP, Dai Z, Gao Z, Lee JH, Ghimire N, Chen J (2017). Detection of sessile serrated adenomas in the proximal colon using wide-field fluorescence endoscopy. Gastroenterology.

[12] Hartmans E, Tjalma JJJ, Linssen MD, Allende PBG, Koller M, Jorritsma-Smit A (2018). Potential red-flag identification of colorectal adenomas with wide-field fluorescence molecular endoscopy. Theranostics.

[13] Nagengast WB, Hartmans E, Garcia-Allende PB, Peters FTM, Linssen MD, Koch M (2019). Near-infrared fluorescence molecular endoscopy detects dysplastic oesophageal lesions using topical and systemic tracer of vascular endothelial growth factor A. Gut.

[14] Maeda H (2012). Macromolecular therapeutics in cancer treatment: the EPR effect and beyond. J Control Release.

[15] Tummers QR, Hoogstins CE, Peters AA, de Kroon CD, Trimbos JB, van de Velde CJ (2015). The value of intraoperative near-infrared fluorescence imaging based on enhanced permeability and retention of Indocyanine Green: feasibility and false-positives in ovarian cancer. PLoS ONE.

[16] Regula J, MacRobert AJ, Gorchein A, Buonaccorsi GA, Thorpe SM, Spencer GM (1995). Photosensitisation and photodynamic therapy of oesophageal, duodenal, and colorectal tumours using 5 aminolaevulinic acid induced protoporphyrin IX—a pilot study. Gut.

[17] Zhao SG, Chen XF, Wang LG, Yang G, Han DY, Teng L (2013). Increased expression of ABCB6 enhances protoporphyrin IX accumulation and photodynamic effect in human glioma. Ann Surg Oncol.

[18] Utsuki S, Oka H, Sato S, Shimizu S, Suzuki S, Tanizaki Y et al (2007) Histological examination of false positive tissue resection using 5-aminolevulinic acid-induced fluorescence guidance. Neurol Med Chir (Tokyo) 47(5):210–3; discussion 3–4 10.2176/nmc.47.210 17527047

[19] Chohan MO, Berger MS (2019). 5-Aminolevulinic acid fluorescence guided surgery for recurrent high-grade gliomas. J Neurooncol.

[20] Vleugels JLA, Rutter MD, Ragunath K, Rees CJ, Ponsioen CY, Lahiff C (2018). Chromoendoscopy versus autofluorescence imaging for neoplasia detection in patients with longstanding ulcerative colitis (FIND-UC): an international, multicentre, randomised controlled trial. Lancet Gastroenterol Hepatol.

[21] Zhao ZY, Guan YG, Li BR, Shan YQ, Yan FH, Gao YJ (2015). Detection and miss rates of autofluorescence imaging of adenomatous and polypoid lesions during colonoscopy: a systematic review and meta-analysis. Endosc Int Open.

[22] Takeuchi Y, Sawaya M, Oka S, Tamai N, Kawamura T, Uraoka T (2019). Efficacy of autofluorescence imaging for flat neoplasm detection: a multicenter randomized controlled trial (A-FLAT trial). Gastrointest Endosc.

[23] Tjalma JJ, Garcia-Allende PB, Hartmans E, Terwisscha van Scheltinga AG, Boersma-vanEk W, Glatz J (2016). Molecular fluorescence endoscopy targeting vascular endothelial growth factor A for improved colorectal polyp detection. J Nucl Med.

[24] Terwisscha van Scheltinga AG, van Dam GM, Nagengast WB, Ntziachristos V, Hollema H, Herek JL (2011). Intraoperative near-infrared fluorescence tumor imaging with vascular endothelial growth factor and human epidermal growth factor receptor 2 targeting antibodies. J Nucl Med.

[25] Linders D, Deken M, van der Valk M, Tummers W, Bhairosingh S, Schaap D (2021). CEA, EpCAM, αvβ6 and uPAR expression in rectal cancer patients with a pathological complete response after neoadjuvant therapy. Diagnostics (Basel).

[26] Boogerd LSF, Hoogstins CES, Schaap DP, Kusters M, Handgraaf HJM, van der Valk MJM (2018). Safety and effectiveness of SGM-101, a fluorescent antibody targeting carcinoembryonic antigen, for intraoperative detection of colorectal cancer: a dose-escalation pilot study. Lancet Gastroenterol Hepatol.

[27] Wu AM, Senter PD (2005). Arming antibodies: prospects and challenges for immunoconjugates. Nat Biotechnol.

[28] Hoetker MS, Goetz M (2013). Molecular imaging in endoscopy. United European Gastroenterol J.

[29] Tjalma JJJ, Koller M, Linssen MD, Hartmans E, de Jongh S, Jorritsma-Smit A (2020). Quantitative fluorescence endoscopy: an innovative endoscopy approach to evaluate neoadjuvant treatment response in locally advanced rectal cancer. Gut.

[30] Nagengast WB (2019) A prospective follow-up intervention study: detection of early esophageal cancer by near-infrared fluoresence molecular endoscopy using bevacizumab-800CW (ESCEND). Identifier NCT03877601. https://clinicaltrials.gov/ct2/show/NCT03877601?cond=Oesophagus+Cancer&cntry=NL&draw=3

[31] Goetz M, Hoetker MS, Diken M, Galle PR, Kiesslich R (2013). In vivo molecular imaging with cetuximab, an anti-EGFR antibody, for prediction of response in xenograft models of human colorectal cancer. Endoscopy.

[32] de Valk KS, Deken MM, Schaap DP, Meijer RP, Boogerd LS, Hoogstins CE (2021). Dose-finding study of a CEA-targeting agent, SGM-101, for intraoperative fluorescence imaging of colorectal cancer. Ann Surg Oncol.

[33] Atreya R, Neumann H, Neufert C, Waldner MJ, Billmeier U, Zopf Y (2014). In vivo imaging using fluorescent antibodies to tumor necrosis factor predicts therapeutic response in Crohn’s disease. Nat Med.

[34] Rath T, Bojarski C, Neurath MF, Atreya R (2017). Molecular imaging of mucosal α4β7 integrin expression with the fluorescent anti-adhesion antibody vedolizumab in Crohn’s disease. Gastrointest Endosc.

[35] Boogerd LSF, Boonstra MC, Prevoo HAJM, Handgraaf HJM, Kuppen PJK, van de Velde CJH (2019). Fluorescence-guided tumor detection with a novel anti-EpCAM targeted antibody fragment: preclinical validation. Surg Oncol.

[36] Vahrmeijer AL (2018) Study for intra-operative imaging of gastrointestinal cancer using VB5–845D-800CW. Identifier NTR7570. https://www.trialregister.nl/trial/7363

[37] Bernhard W, El-Sayed A, Barreto K, Gonzalez C, Fonge H, Geyer CR (2019). Near infrared imaging of epidermal growth factor receptor positive xenografts in mice with domain I/II specific antibody fragments. Theranostics.

[38] de Valk KS, Deken MM, Handgraaf HJM, Bhairosingh SS, Bijlstra OD, van Esdonk MJ (2020). First-in-human assessment of cRGD-ZW800-1, a zwitterionic, integrin-targeted, near-infrared fluorescent peptide in colon carcinoma. Clin Cancer Res.

[39] de Jongh SJ, Voskuil FJ, Schmidt I, Karrenbeld A, Kats-Ugurlu G, Meersma GJ (2020). C-Met targeted fluorescence molecular endoscopy in Barrett’s esophagus patients and identification of outcome parameters for phase-I studies. Theranostics.

[40] Chen J, Jiang Y, Chang TS, Joshi B, Zhou J, Rubenstein JH (2021). Multiplexed endoscopic imaging of Barrett’s neoplasia using targeted fluorescent heptapeptides in a phase 1 proof-of-concept study. Gut.

[41] Yim JJ, Harmsen S, Flisikowski K, Flisikowska T, Namkoong H, Garland M (2021). A protease-activated, near-infrared fluorescent probe for early endoscopic detection of premalignant gastrointestinal lesions. Proc Natl Acad Sci U S A.

[42] van Dam GM, Themelis G, Crane LM, Harlaar NJ, Pleijhuis RG, Kelder W (2011). Intraoperative tumor-specific fluorescence imaging in ovarian cancer by folate receptor-α targeting: first in-human results. Nat Med.

[43] Messmann H, Knüchel R, Bäumler W, Holstege A, Schölmerich J (1999). Endoscopic fluorescence detection of dysplasia in patients with Barrett’s esophagus, ulcerative colitis, or adenomatous polyps after 5-aminolevulinic acid-induced protoporphyrin IX sensitization. Gastrointest Endosc.

[44] Maranda EL, Heifetz R, Estes WA, Cortizo J, Shareef S, Jimenez JJ (2016). Porphyria and vampirism—a myth, sensationalized. JAMA Dermatol.

[45] Harmsen S, Rogalla S, Huang R, Spaliviero M, Neuschmelting V, Hayakawa Y (2019). Detection of premalignant gastrointestinal lesions using surface-enhanced resonance Raman scattering-nanoparticle endoscopy. ACS Nano.

[46] Rogalla S, Flisikowski K, Gorpas D, Mayer AT, Flisikowska T, Mandella MJ (2019). Biodegradable fluorescent nanoparticles for endoscopic detection of colorectal carcinogenesis. Adv Funct Mater.

[47] Frangioni JV (2003). In vivo near-infrared fluorescence imaging. Curr Opin Chem Biol.

[48] Mortensen O, Nerup N, M T, Svendsen M, Shiwaku H, Achiam M (2020) Fluorescence guided intraluminal endoscopy in the gastrointestinal tract: a systematic review. World J Gastrointest Endosc 12(10):388-400 10.4253/wjge.v12.i10.388 PMC7579525 33133375

[49] Shrivastav M, Gounaris E, Khan MW, Ko J, Ryu SH, Bogyo M (2018). Validation of near infrared fluorescence (NIRF) probes in vivo with dual laser NIRF endoscope. PLoS ONE.

[50] Weissleder R, Ntziachristos V (2003). Shedding light onto live molecular targets. Nat Med.

[51] Haque A, Faizi MSH, Rather JA, Khan MS (2017). Next generation NIR fluorophores for tumor imaging and fluorescence-guided surgery: a review. Bioorg Med Chem.

[52] American Society for Gastrointestinal Endoscopy (2014). Technology Committee. Confocal Laser Endomicroscopy Gastrointest Endosc.

[53] Rogalla S, Contag CH (2015). Early cancer detection at the epithelial surface. Cancer J.

[54] Upadhyay R, Sheth RA, Weissleder R, Mahmood U (2007). Quantitative real-time catheter-based fluorescence molecular imaging in mice. Radiology.

[55] Joshi BP, Duan X, Kwon RS, Piraka C, Elmunzer BJ, Lu S (2016). Multimodal endoscope can quantify wide-field fluorescence detection of Barrett’s neoplasia. Endoscopy.

[56] Hoy CL, Gamm UA, Sterenborg HJ, Robinson DJ, Amelink A (2013). Method for rapid multidiameter single-fiber reflectance and fluorescence spectroscopy through a fiber bundle. J Biomed Opt.

[57] de Jongh SJ, Vrouwe JPM, Voskuil FJ, Schmidt I, Westerhof J, Koornstra JJ (2020). The optimal imaging window for dysplastic colorectal polyp detection using c-Met-targeted fluorescence molecular endoscopy. J Nucl Med.

[58] Zhang HZ, Jin GF, Shen HB (2012). Epidemiologic differences in esophageal cancer between Asian and Western populations. Chin J Cancer.

[59] Levine DS, Blount PL, Rudolph RE, Reid BJ (2000). Safety of a systematic endoscopic biopsy protocol in patients with Barrett’s esophagus. Am J Gastroenterol.

[60] Peters FP, Curvers WL, Rosmolen WD, de Vries CE, Ten Kate FJ, Krishnadath KK (2008). Surveillance history of endoscopically treated patients with early Barrett’s neoplasia: nonadherence to the Seattle biopsy protocol leads to sampling error. Dis Esophagus.

[61] Abrams JA, Kapel RC, Lindberg GM, Saboorian MH, Genta RM, Neugut AI et al (2009) Adherence to biopsy guidelines for Barrett’s esophagus surveillance in the community setting in the United States. Clin Gastroenterol Hepatol 7(7):736–42; quiz 10 10.1016/j.cgh.2008.12.027 PMC3139243 19268726

[62] Wani S, Williams JL, Komanduri S, Muthusamy VR, Shaheen NJ (2019). Endoscopists systematically undersample patients with long-segment Barrett’s esophagus: an analysis of biopsy sampling practices from a quality improvement registry. Gastrointest Endosc.

[63] Lynch HT, Snyder CL, Shaw TG, Heinen CD, Hitchins MP (2015). Milestones of Lynch syndrome: 1895–2015. Nat Rev Cancer.

[64] Nadeem MS, Kumar V, Al-Abbasi FA, Kamal MA, Anwar F (2020). Risk of colorectal cancer in inflammatory bowel diseases. Semin Cancer Biol.

[65] Nagorni A, Bjelakovic G, Petrovic B (2012). Narrow band imaging versus conventional white light colonoscopy for the detection of colorectal polyps. Cochrane Database Syst Rev.

[66] Bisschops R, Bessissow T, Joseph JA, Baert F, Ferrante M, Ballet V (2018). Chromoendoscopy versus narrow band imaging in UC: a prospective randomised controlled trial. Gut.

[67] Ferrari L, Fichera A (2015). Neoadjuvant chemoradiation therapy and pathological complete response in rectal cancer. Gastroenterol Rep (Oxf).

[68] Park IJ, You YN, Agarwal A, Skibber JM, Rodriguez-Bigas MA, Eng C (2012). Neoadjuvant treatment response as an early response indicator for patients with rectal cancer. J Clin Oncol.

[69] Maas M, Beets-Tan RG, Lambregts DM, Lammering G, Nelemans PJ, Engelen SM (2011). Wait-and-see policy for clinical complete responders after chemoradiation for rectal cancer. J Clin Oncol.

[70] Renehan AG, Malcomson L, Emsley R, Gollins S, Maw A, Myint AS (2016). Watch-and-wait approach versus surgical resection after chemoradiotherapy for patients with rectal cancer (the OnCoRe project): a propensity-score matched cohort analysis. Lancet Oncol.

[71] Soler D, Chapman T, Yang LL, Wyant T, Egan R, Fedyk ER (2009). The binding specificity and selective antagonism of vedolizumab, an anti-alpha4beta7 integrin therapeutic antibody in development for inflammatory bowel diseases. J Pharmacol Exp Ther.

[72] Nagengast WB (2020) NIR FME of labelled vedolizumab to elucidate the mechanism of action and predicting response in IBD patients (VISION). Identifier NCT04112212. https://clinicaltrials.gov/ct2/show/NCT04112212

[73] Lee HS, Cleynen I (2019). Molecular profiling of inflammatory bowel disease: is it ready for use in clinical decision-making?. Cells.

[74] Al-Mustanjid M, Mahmud SMH, Royel MRI, Rahman MH, Islam T, Rahman MR (2020). Detection of molecular signatures and pathways shared in inflammatory bowel disease and colorectal cancer: a bioinformatics and systems biology approach. Genomics.

[75] Yim JJ, Tholen M, Klaassen A, J. S, Bogyo M (2018) Optimization of a protease activated probe for optical surgical navigation. Mol Pharm 5(3):750–8 10.1021/acs.molpharmaceut.7b00822 29172524

[76] Spadaccini M, Iannone A, Maselli R, Badalamenti M, Desai M, Chandrasekar VT (2021). Computer-aided detection versus advanced imaging for detection of colorectal neoplasia: a systematic review and network meta-analysis. Lancet Gastroenterol Hepatol.

[77] Goyal H, Mann R, Gandhi Z, Perisetti A, Ali A, Aman Ali K (2020). Scope of artificial intelligence in screening and diagnosis of colorectal cancer. J Clin Med.

[78] van der Sommen F, de Groof J, Struyvenberg M, van der Putten J, Boers T, Fockens K (2020). Machine learning in GI endoscopy: practical guidance in how to interpret a novel field. Gut.

[79] Cohen-Mekelburg S, Berry S, Stidham RW, Zhu J, Waljee AK (2021). Clinical applications of artificial intelligence and machine learning-based methods in inflammatory bowel disease. J Gastroenterol Hepatol.

[80] Gubatan J, Levitte S, Patel A, Balabanis T, Wei MT, Sinha SR (2021). Artificial intelligence applications in inflammatory bowel disease: emerging technologies and future directions. World J Gastroenterol.

[81] Glissen Brown JR, Mansour NM, Wang P, Gross SA, Sengupta N, Berzin TM (2021) Deep learning computer-aided polyp detection reduces adenoma miss rate: a U.S. multi-center randomized tandem colonoscopy study (Cadet-CS Trial). Clin Gastroenterol Hepatol (9–14):Online available: https://www.sciencedirect.com/science/article/pii/S1542356521009733?via%3Dihub 10.1016/j.cgh.2021.09.009 34530161

[82] Wu L, Shang R, Sharma P, Zhou W, Liu J, Yao L (2021). Effect of a deep learning-based system on the miss rate of gastric neoplasms during upper gastrointestinal endoscopy: a single-centre, tandem, randomised controlled trial. Lancet Gastroenterol Hepatol.

